# Do Iron Oxide Nanoparticles Have Significant Antibacterial Properties?

**DOI:** 10.3390/antibiotics10070884

**Published:** 2021-07-20

**Authors:** Sergey V. Gudkov, Dmitriy E. Burmistrov, Dmitriy A. Serov, Maksim B. Rebezov, Anastasia A. Semenova, Andrey B. Lisitsyn

**Affiliations:** 1 Prokhorov General Physics Institute of the Russian Academy of Sciences, 119991 Moscow, Russia; dmitriiburmistroff@gmail.com (D.E.B.); dmitriy_serov_91@mail.ru (D.A.S.); rebezov@yandex.ru (M.B.R.); 2 V.M. Gorbatov Federal Research Center for Food Systems of the Russian Academy of Sciences, 109316 Moscow, Russia; a.semenova@fncps.ru (A.A.S.); info@fncps.ru (A.B.L.)

**Keywords:** nanoparticles, iron oxide, antimicrobial effect, green synthesis

## Abstract

The use of metal oxide nanoparticles is one of the promising ways for overcoming antibiotic resistance in bacteria. Iron oxide nanoparticles (IONPs) have found wide applications in different fields of biomedicine. Several studies have suggested using the antimicrobial potential of IONPs. Iron is one of the key microelements and plays an important role in the function of living systems of different hierarchies. Iron abundance and its physiological functions bring into question the ability of iron compounds at the same concentrations, on the one hand, to inhibit the microbial growth and, on the other hand, to positively affect mammalian cells. At present, multiple studies have been published that show the antimicrobial effect of IONPs against Gram-negative and Gram-positive bacteria and fungi. Several studies have established that IONPs have a low toxicity to eukaryotic cells. It gives hope that IONPs can be considered potential antimicrobial agents of the new generation that combine antimicrobial action and high biocompatibility with the human body. This review is intended to inform readers about the available data on the antimicrobial properties of IONPs, a range of susceptible bacteria, mechanisms of the antibacterial action, dependence of the antibacterial action of IONPs on the method for synthesis, and the biocompatibility of IONPs with eukaryotic cells and tissues.

## 1. Introduction

Nowadays, the application of nanotechnological solutions, such as the use of nanoparticles, is one of the promising ways to overcome antibiotic resistance in bacteria [1,2,3,4,5].

Nanoparticles (NPs) of several metals and their oxides, such as Ag, ZnO, Fe_2_O_3_, Fe_3_O_4_, Al_2_O_3_, TiO_2_, and CuO, exert antibacterial action against Gram-negative and Gram-positive bacteria, as well as the antifungal action [6,7,8,9,10,11,12,13,14].

Iron is one of the most abundant elements on Earth and the fourth-most abundant element in the Earth’s crust. Iron makes up more than 85% of the mass of the Earth’s core and about 5% of the mass of the Earth’s crust [15,16]. In living systems, iron is one of the key microelements. It has several important functions: it is a cofactor of several enzymes (catalase) and transport proteins (hemoglobin), ETC proteins (cytochromes and FeS proteins), and is necessary for DNA repair [17,18,19,20]. Iron is also found in the regulatory proteins of enterobacteria *Salmonella enterica*, including Fur, Fnr, NorR, SoxR, IscR, and NsrR [21,22,23,24,25,26]. Several bacteria can accumulate iron oxides in special organelles called magnetosomes—for example, *Magnetospirillum magneticum* [27]. It is assumed that they provide bacteria with the constant magnetic dipole, presumably for navigation purposes [28]. It was shown in *Magnetospirillum magneticum* wild-type and DmagA1-/- that magnetosomes plays a key role in magneto-aerotaxis. Magneto-aerotaxis is the direct motion of bacteria downward in microaerobic environments favorable to growth [29]. Bacteria that use the iron oxidation reaction Fe^2+^ + 0.25 O_2_ + H^+^ → Fe^3+^ + 0.5 H_2_O for energy generation and metabolism maintenance have been described. A minimum two groups of obligate iron-oxidizing bacteria: *Betaproteobacteria* and *Zetaproteobacteria* are described in the phylum *Proteobacteria* [30]. Iron is necessary for the proliferation of microbial agents of infectious diseases that developed ways for iron acquisition from the host, while the host has protective mechanisms preventing iron acquisition by microorganisms [30,31].

In light of the presented facts, it is logical to ask whether NPs (IONPs) based on compounds of the biogenic element, which is so important for vital activities, can have a bactericidal effect. On the one hand, a negative answer is expected; however, several studies noticed the antimicrobial action of IONPs [32,33]. On the other hand, the bactericidal action was repeatedly confirmed for NPs based on other biogenic elements: ZnO and CuO, as was mentioned above.

Iron is like a double-edged sword. Despite its above-mentioned functions in living organisms, it is able to catalyze reactions of damaging DNA, lipids, and proteins by the Fenton reaction [34]. In this reaction, the free Fe^2+^ ion reacts with hydrogen peroxide (H_2_O_2_); as a result, a hydroxyl radical and Fe^3+^ ion are formed. The following reaction of Fe^3+^ with the superoxide anion radical (O_2_^−^^∙^) leads to the formation of molecular oxygen (O_2_) and regeneration of Fe^2+^ as the initial catalyst. To protect it from the damage caused by the generation of hydroxyl radicals, it is necessary to maintain an extremely low level of free iron ions inside cells [35]. ROS generation is no single mechanism of antibacterial action of IONPs. A more detail description of these mechanisms is contained in Section 2.2.

The antibacterial properties are found both in nanoparticles based on iron oxides (IONPs) and in free iron ions; however, contrary to free ions, IONPs do not exert a significant toxic effect on mammalian cells [8,36,37]. Iron oxide nanoparticles can be obtained by different methods, from laser ablation to chemical synthesis [38,39,40,41,42,43]. It is assumed that the antibacterial properties of iron oxide nanoparticles are associated not only with the oxide form but, also, with the size, morphology, and other physicochemical properties of nanoparticles. Several types of iron oxides are known. The most frequently found are hematite Fe_2_O_3_, magnetite Fe_3_O_4_, and limonite Fe_2_O_3_ × H_2_O [5,16].

Iron oxide nanoparticles (IONPs) have found wide applications in different fields of biomedicine—for example, in visualization and diagnostics [44]; in magnetic resonance imaging and computed tomography [45,46,47,48]; in positron emission tomography [49]; in cancer therapy with magnetic hyperthermia [50,51,52]; and for the separation of cells or molecules and the development of biosensors, which can applied to immunoassays, neuroelectronic studies, and biomedical imaging [53,54,55,56]. IONPs may be used in the imaging and tracking of brain cells in vivo [57]. A possibility of using iron oxide nanoparticles for delivering medicines and viral vectors to target cells is shown [58,59]. The antibacterial activity of iron oxide nanoparticles (IONPs) is of special interest, as the emergence of antibiotic-resistant strains is a serious problem for world public health. The direct bactericidal action of IONPs was described by the example of *S. aureus* [32]. Fe_3_O_4_ NPs can be used in regenerative medicine [60]. With that, IONPs have good biocompatibility in vivo and in vitro [61,62], which qualitatively distinguish IONPs from ZnO NPs having high cytotoxicity [63,64]. The balance of the antimicrobial activity and biocompatibility makes IONPs an attractive candidate for the role of an antimicrobial preparation of the new generation. The present review is intended to inform readers about available data on the antibacterial properties of IONPs, a range of susceptible bacteria, mechanisms of the antibacterial action, the dependence of the antibacterial action of IONPs on the method for synthesis, and the biocompatibility of IONPs with eukaryotic cells and tissues.

## 2. Main Part

### 2.1. Susceptible Microorganisms

A list of microorganisms susceptible to the toxic action of IONPs is presented in Table 1. A minimum of 10 species of Gram-negative and 11 species of Gram-positive bacteria, as well as three fungal species susceptible to IONPs, have been mentioned in the literature (Table 1). The majority of the indicated microorganisms have epidemiological significance [65]. A range of the bacteriostatic concentrations for IONPs is quite wide and makes up 25–2000 µg/mL.

IONPs have antimicrobial activity against both Gram-positive (including *Staphylococcus aureus*) and Gram-negative (including *Escherichia coli*) bacteria [33]. The data about the dependence of the antibacterial action of IONPs on the bacterial group (Gram-positive or Gram-negative) are ambiguous. On the one hand, there are data about the comparable effects of IONPs against Gram-negative and Gram-positive bacteria [91], similar to CuO [92], which distinguishes IONPs from ZnO NPs [93]. On the other hand, there are data about the more pronounced bacteriostatic action of Fe_3_O_4_ against Gram-negative bacteria compared to Gram-positive [66]. The authors linked the indicated differences with the peculiarities of the cell wall structure and metabolism of Gram-positive and Gram-negative bacteria [66].

### 2.2. The Mechanisms of Antibacterial IONP Activity

**Figure 1:**
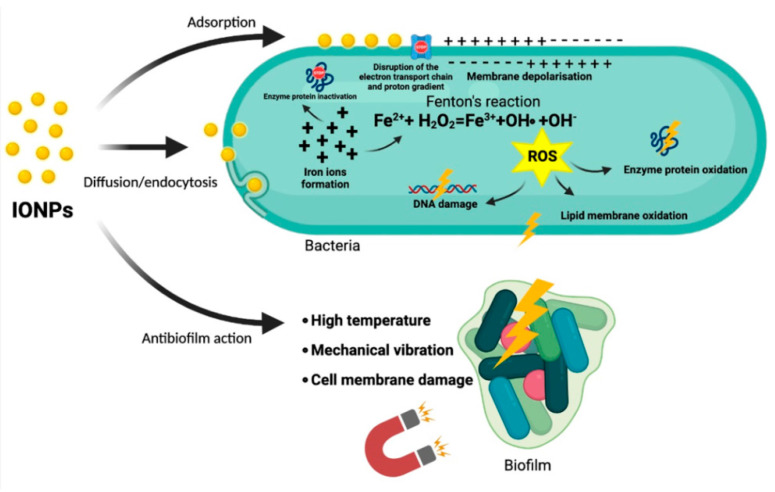
The mechanisms of IONP antibacterial activity.

One of the main mechanisms of IONP toxicity is ROS generation [5,94], including in photocatalysis, Fenton reactions, or similar ones [88]. ROS, in turn, have a genotoxic action, damaging DNA molecules (Figure 1) [94]. An increase in ROS concentration can be caused by a decrease in the activity of antioxidant system enzymes (SOD, catalase, and glutathione reductase) [67]. Metal ions are able to bind mecapto (–SH), amino (–NH), and carboxyl (–COOH) groups of proteins, including enzymes, which leads to inactivation or partial inhibition [95]. Additionally, IONPs damage the bacterial cell wall integrity, as shown in reference [94]. The direct binding of IONPs with the cell wall of *Staphylococcus aureus* was shown by scanning electron microscopy [96]. IONPs can cause a decrease in the expression of antibiotic resistance genes (ARGs) in antibiotic-resistant bacteria found in operating rooms [5]. IONPs are able to disturb the function of F_0_/F_1_-ATPase and reduce the rate of H^+^ flow through the membrane and the redox potential [66]. The mechanisms of the antimicrobial action for IONPs have been suggested in several studies based on their size and are common for other types of metal oxide nanoparticles [95,97]. An ability to inhibit DNA replication by the inactivation of topoisomerase is described for nanoparticles with small sizes [98]. It was shown by the method of electron microscopy that Fe_2_O_3_ NPs can bind directly with the cell wall of *E. coli*. IONPs can also penetrate into the cytoplasm, concentrate in it, and cause vacuole formation and cell wall disruption [84,99].

Fe_3_O_4_ IONPs can concentrate between the outer and inner membranes of the cell wall in Gram-negative bacteria due to binding with the FHL complex in the inner membrane. Therefore, Fe_3_O_4_ IONPs have more pronounced antimicrobial actions against Gram-negative bacteria [66].

Bactericidal and antibiofilm activities were shown in Fe_3_O_4_ IONPs. Positively charged and neutral IONPs promoted a higher reduction of *Streptococcus mutans* biofilms compared with negatively charged IONPs [89]. IONPs coated with oleic acid can prevent biofilm formation by *S. aureus* and *P. aeruginosa* [85]. IOPNs have the ability to adsorb and penetrate into bacterial biofilms due to their physicochemical characteristics, such as a surface charge, hydrophobicity, and high surface area ratio by volume [100,101].

Iron oxide nanoparticles have both magnetic and paramagnetic properties [68,87,102]. Fe_3_O_4_ NPs with high paramagnetic activity are also named superparamagnetic iron oxide nanoparticles (SPIONs) [103,104]. SPIONs in the presence of the alternating magnetic fields cause cell death and biofilm destruction due to the vibration damage, local hyperthermia, and ROS generation. All of the above-mentioned factors lead to the dissociation of bacteria from a biofilm, damage of the bacterial cell wall, membrane rupture, the fusion of different cells with each other, and death [69].

In 80% of studies, IONPs show only bacteriostatic action. The bactericidal action of IONPs is described in the literature in 20% of cases.

### 2.3. Methods of IOPNs Synthesis

The methods for IONP synthesis are multiple and include coprecipitation [105], thermal decomposition [70], low temperature synthesis [71], the sol–gel method [106], hydrothermal method [69], electrochemical method [83], laser ablation [91,107], sonochemical, microwave, microemulsion methods, matrix-mediated method using PVA, “green synthesis” [68], and many others [32,84,108]. In the case of research of IONP antibacterial effects, most used in coprecipitation, thermal decomposition, the sol–gel method, laser ablation, and “green synthesis” (Table 2); therefore, we shall briefly describe these methods below. 

Aqueous coprecipitation is the most widely used chemical method of IONP synthesis [105,109]. In this method, IOPNs are synthesized by the simultaneous precipitation of Fe^2+^ and Fe^3+^ salts (molar ratio 1:2) in a basic solution at room temperature or under heat [105,109,110]. The advantage of the coprecipitation method is the low cost of IOPNs synthesis. It is important in cases of large-scale production [27]. The disadvantages of the method are the large size distribution of produced IONPs, aggregation, poor crystallinity, a high possibility of oxidation, and poor magnetic property [111]. The change of pH in the solution can improve the properties of IONPs synthesized by coprecipitation [112].

The thermal decomposition is a nonaqueous synthesis in which organometallic compounds such as Fe(Acac)_3_, Fe(C_2_O_4_) *×* 2 H_2_O, Fe(CH_3_COO)_2_, or ferrocene suffer decay at high temperatures in organic solvents (high boiled) or via being solvent-free in the presence of stabilizing surfactants like aliphatic amine and fatty acids [113]. This method may generate high-quality IONPs with close distributions of particle sizes and a high magnetism and degree of crystallinity [113]. Addition advantages of this method are the high yield and absence of IONP aggregation [114]. The main disadvantage of this method is the insolubility of produced IONPs in water. Therefore, further steps are required to make their surfaces hydrophilic and use IONPs in biological solutions [115].

The sol–gel method (wet–chemical method) is a sum of reactions of condensation and hydrolysis between iron alkoxides and salts (e.g., chlorides, nitrates, and acetates) [116]. The main advantage of this method is a good homogeneity and size and high purity and quantity of IONPs [116]. The disadvantages of the method are the requirements for compliance with exact values of the pH, temperature, and concentration of the reagents during a synthesis; high cost of precursors; and low wear resistance of synthesized IONPs [117].

Laser ablation synthesis in a solution is a synthesis that is triggered by the immersion of pulsed laser beams on the target material in a liquid solution [118]. Laser ablation synthesis allows to work with a wide range of materials and solvents. The size and clustering of IONPs are difficult to control [118]. Laser ablation allows the synthesis of FeOx crystal to a few atom clusters in the following modification: phosphonates as an aqueous solution and bulk iron as a target [72].

The so-called “green synthesis” has aroused considerable interest. It is a modification of synthesis methods (as a rule, coprecipitation) with the application of plant extracts used as a reducing agent. There are reports about the application of leaf extracts of the *Psidium guajava* [68], *Cynometra ramiflora* [88], *Sida cordifolia* [119], *Zea mays* [87], *Argemone mexicana* [73], *Couroupita guianensis* [81], *Tridax procumb* [120], peel extracts of *Punica granatum* [70], *Ruellia tuberosa* [74], *Malva sylvestris* [82], and *Citrus sinensis* [121]. This method is low-cost, if coprecipitation is used as a basic technique [74,82,121].

Large-scale synthesis is a modification of the coprecipitation method with controlled heating and addition polyacrilic acid salts or sodium oleate as the surfactant [103]

The hydrothermal method is a synthesis of IONPs from iron precursors at high pressure and temperature conditions in an aqueous medium [103,104]. Aqueous synthesis methods generate particles with low crystallization [122]. Replacing water with other organic solvents allows the formation of IONPs with high crystallinity and controlled shapes. This method is named solvothermal synthesis [113]. The disadvantage of this method is the long time it takes for synthesis (hours to days) [123].

### 2.4. Dependence of the Antimicrobial Action of IONPs on the Size and Type of Iron Oxides

**Figure 2:**
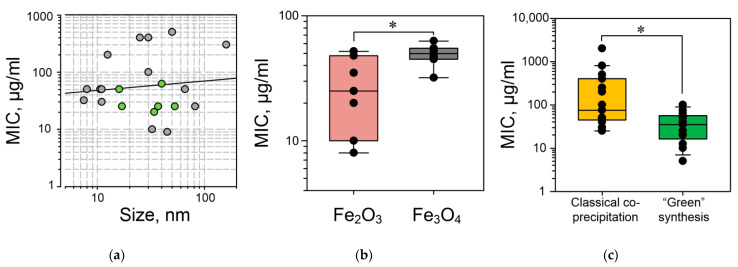
(**a**) Assessment of the dependence of the IONP MIC against *E. coli* on the IONP size. (**b**) Assessment of the dependence of the IONP MIC against *E. coli* on the iron oxide type. (**c**) Assessment of the dependence of the IONP MIC against *E. coli* in the synthesis method. Values of the minimum inhibitory concentrations are taken from the sources in Table 2. In (**a**), the grey color shows the values for the IONPs obtained without “green synthesis”, and the green color shows the values for the IONPs obtained by “green synthesis”. *—*p* < 0.05 by the Mann–Whitney *U* test.

The majority of studied IONPs have a spherical shape (Table 2), which excludes a contribution of the shape into the antimicrobial action. Therefore, we assessed the sizes and compositions of IONPs. Based on the analyzed literature data, we did not reveal an association between the IONPs’ size and the minimum bacteriostatic concentrations (Figure 2a). Several IONP types are distinguished depending on the oxide on which basis they are synthesized: NPs based on hematite (α-Fe_2_O_3_) [5,68], β-Fe_2_O_3_, γ-Fe_2_O_3_, ε-Fe_2_O_3_ [124,125,126], and Fe_3_O_4_ [83,127]. We found that Fe_2_O_3_ NPs show more pronounced bacteriostatic actions compared to Fe_3_O_4_ NPs (Figure 2b). For more detailed analyses, we assessed the contribution of a method for IONP synthesis of their antimicrobial properties. 

### 2.5. Dependence of the Antimicrobial Action of IONPs on a Synthesis Method

The methods for IONPs synthesis are multiple and include coprecipitation [105], thermal decomposition [70], low-temperature synthesis [71], the sol–gel method [106], hydrothermal method [69], electrochemical method [83], laser ablation [91,107], sonochemical, microwave, microemulsion methods, matrix-mediated method using PVA, and many others [32,84,108]. IONPs synthesized by the low-temperature method from iron sulfate showed an antimicrobial effect against *E. coli*, *P. aeruginosa*, *Serratia marcescens*, and *Listeria monocytogenes*, exerting bacteriostatic action and inhibiting biofilm formation [71]. NPs obtained by laser ablation had comparable bacteriostatic effects against Gram-negative (*Escherichia coli*, *Pseudomonas aeruginosa,* and *Serratia marcescens*) and Gram-positive (*Staphylococcus aureus*) bacteria. The bacteriostatic actions of IONPs do not depend on a solvent (SDS or DMF) or bacterial group (Gram-positive or Gram-negative) [91].

#### 2.5.1. Coprecipitation Method

The most common method for IONP synthesis when studying the antimicrobial properties is the coprecipitation of salts Fe^3+^/Fe^2+^ [56,73,75,86,127,128,129]. This synthesis method is the most available. Modifications of the method are possible. For instance, the addition of oleic acid for the generation of conjugated IONPs [127,129], as well as the coprecipitation of different metal salts, allow us to obtain composite NPs—for example, based on FeSO_4_ × 7 H_2_O and Co(NO_3_)_2_ × 6 H_2_O [76]. One of the methods for improving the antimicrobial properties of IONPs is the use of composites—for example, α-Fe_2_O_3_/Co_3_O_4_ [105]. Composite NPs have more pronounced antimicrobial actions against *B. subtilis, S. aureus, E.coli*, and *S. typhimirium*. The synergistic effect of Fe_2_O_3_ and Co_3_O_4_ was observed compared to oxides used individually. However, upon the strong bacteriostatic action (practically a full inhibition of the bacterial growth at a concentration of 1200 mg/mL), the bactericidal action was almost absent [76]. α-Fe_2_O_3_/ZnO NPs show more pronounced bacteriostatic actions against Gram-positive *Bacillus subtilis* and *Staphylococcus aureus* and Gram-negative *Escherichia coli* and *Salmonella typhi* than IONPs and ZnO NPs; with that, the size of the inhibition zone increases when the ZnO concentration in the composite is increased [76], (Table 2).

Compared to Fe_3_O_4_ NPs, the composite Fe_3_O_4_/SiO_2_ NPs has a more pronounced photocatalytic bactericidal action against *Escherichia coli* and *Staphylococcus aureus*; with that, the effect was higher against Gram-positive bacteria [130]. The use of the combined method for IONPs synthesis allows achieving a significant bacteriostatic effect against *Staphylococcus aureus*, *Xanthomonas*, *Escherichia coli*, and *Proteus vulgaris* [83].

#### 2.5.2. “Green Synthesis”

The so-called “green synthesis” has aroused considerable interest. It is a modification of synthesis methods (as a rule, coprecipitation) with the application of plant extracts used as a reducing agent [83,106,107,108,109,110].

IONPs synthetized by the “green” method show comparable antimicrobial effects against both Gram-negative (*E. coli*) and Gram-positive (*S. aureus*) bacteria [68]. However, the antimicrobial effect of 50–100 µg/µL of IONPs is about three times lower than that of 20 µg/mL of streptomycin. IONPs synthesized in the presence of *Punica granatum* peel extract exert a bacteriostatic effect on *Pseudomonas aeruginosa*; with that, these IONPs do not have hemolytic activity against erythrocytes [70]. IONPs in complex with *Cynometra ramiflora* extract have more pronounced bacteriostatic effects against Gram-positive *S. epidermalis* compared to Gram-negative *E. coli* [88]. NPs synthesized in the medium of the *Zea mays* extract did not have their own antimicrobial and antifungal properties but significantly enhanced the bacteriostatic action of kanamycin and rifampicin against Gram-positive *Bacillus cereus*, *Listeria monocytogenes*, and *Staphylococcus aureus* and Gram-negative *Escherichia coli* and *Salmonella typhimurium*, as well as the antifungal activity these antibiotics against six strains of *Candida* [87]. In addition to the antimicrobial properties, IONPs obtained as a result of “green” synthesis had antioxidant properties and inhibited their proteasome activity, which allowed us to regard IONPs as possible candidates for cancer therapy [87].

Compared to Fe_3_O_4_ NPs, Fe_3_O_4_/*Malva sylvestris* NPs had more pronounced bacteriostatic and bactericidal effects against *Staphylococcus aureus*, *Corynebacterium* sp., *Pseudomonas aeruginosa*, and *Klebsiella pneumoniae* and exerted cytotoxic action against the Hep-G2 and MCF-7 cell lines [82].

IONPs synthesized in the *Argemone mexicana* extract had more pronounced bacteriostatic activity against *E. coli*, *P. mirabilis*, and *B. subtilis* than pure IONPs, which was comparable with the effects of streptomycin [73].

Fe_3_O_4_ NPs synthesized with a *Couroupita guianensis* extract inhibited the growth of *E. coli*, *S. typhimurium*, *K. pneumoniae*, and *S. aureus* and induced the apoptosis of the hepatocellular carcinoma (HepG2) cell line [81]. IONPs synthesized in a *Ruellia tuberosa* extract inhibited the growth of *E. coli*, *K. pneumoniae*, and *S. aureus* in a dose-dependent manner. The IONP effectiveness turned to be higher than that of streptomycin. The mechanism of antimicrobial action is the photocatalytic generation of ROS [74]. Fe_2_O_3_*/Citrus sinensis* NPs exerted a comparable bacteriostatic action against Gram-positive (*B. subtilis* and *S. aureus*) and Gram-negative (*E. coli* and *P. aeruginosa*) bacteria. The inhibitory effect of Fe_2_O_3_*/Citrus sinensis* NPs was comparable with chlorhexidine, hexachlorophene, benzalkonium chloride, and phenol taken in equal concentrations [121]. α-Fe_2_O_3_ NPs, in combination with a *Sida cordifolia* extract, had comparable bacteriostatic activity against *E. coli*, *K. pneumoniae*, *B. subtilis*, and *S. aureus*. The bacteriostatic effect against Gram-positive bacteria was more strongly pronounced and was comparable with the effect of neomycin [119]. Unfortunately, for several extracts—for example, *Couroupita guianensis*—“green synthesis” leads to an enhancement of IONP cytotoxicity [81]. In another study, the antioxidant properties were described for Fe_3_O_4_ NPs synthesized by the “green method” [87]. In a meta-analysis, we found that IONPs generated by the “green synthesis” method had three times more pronounced bacteriostatic activity than IONPs generated by the coprecipitation method (Figure 2c).

### 2.6. Additional Methods for Increasing the Antimicrobial Activity of IONPs

Iron oxide nanoparticles have both magnetic and paramagnetic properties [68,87,102,103,104]. The use of an alternating magnetic field allows additional increases in the bactericidal action of Fe_3_O_4_ NPs against *E. coli* and *S. aureus*, causing cell death and biofilm destruction due to the photocatalytic generation of ROS, and local hyperthermia and vibration damage occurred under the action of the magnetic field. All of the above-mentioned factors lead to the dissociation of bacteria from the biofilm, damage of the bacterial cell wall, membrane rupture, the fusion of different cells with each other, and death [69].

Fe_2_O_4_ composite NPs with the addition of different ratios of Co and Mn have magnetic properties due to Fe_2_O_4_ and inhibit the growth of *E. coli* and cause damage to *E. coli* and *B. subtilis* in a dose-dependent manner [102].

IONP conjugation with carbon nanotubes allows achieving a bactericidal effect against Gram-negative (*E. coli* and *K. pneumoniae*) and Gram-positive (*Staphylococcus aureus*) bacteria; with that, the CFU were reduced by two and more times compared to the control [77]. Carbon nanotubes/IONPs accelerated wound healing in mice in a wound-healing test by 25% and 50% compared to IONPs or carbon nanotubes taken individually. It is worth noting that, in this study, the size of the inhibition zone increased insignificantly upon a considerable decrease in the CFU; therefore, the antimicrobial effect of IONPs assessed by a size of the inhibition zone in the majority of studies can be underestimated. In contrast to other IONPs types, Fe_3_O_4_ IONPs coated with oleic acid exert a different effect on the growth and viability of Gram-positive (*Enterococcus hirae*) and Gram-negative (*E. coli*) bacteria. More pronounced antimicrobial action was observed against Gram-negative bacteria [127]. The authors linked this phenomenon with differences in the cell wall structure; in particular, with the ability of Fe_3_O_4_ IONPs to concentrate between the outer and inner membranes of the cell wall in Gram-negative bacteria and the presence of the FHL complex in the inner membrane of *E. coli*, which is an additional target for IONP Fe_3_O_4_. Fe_3_O_4_ NPs covered with oleic acid cause a reduction in the growth of kanamycin- and ampicillin-resistant *E. coli* strains due to retardation of the logarithmic growth phase, lag phase extension, reduction of the H^+^ flow through the membrane, and redox potential [85,131]. IONPs coated with oleic acid not only inhibit the growth of *S. aureus* and *P. aeruginosa* but also prevent biofilm formation [131]. 

Surface modification is also a key way to improve IONP the antibacterial properties [78] The conjugation of IONPs with chitosan enhanced the bactericidal action of IONPs against *Bacillus subtilis* and *Escherichia coli* due to ROS generation [78].

Fe_3_O_4_ NPs covered with polyethylene glycol (PEG) exert a dose-dependent bactericidal action against the *E. coli* and *S. aureus* and antibiotic-resistant *Micrococcus luteus* strain. The mechanism of toxicity resides in a decrease in the activity of the antioxidant system enzymes (SOD, catalase, and glutathione reductase) and, as a consequence, enhancement of ROS generation and lipid oxidation [67].

Fe_3_O_4_ NPs conjugated with chitosan have bactericidal and fungicidal actions against *Candida albicans*, *Aspergillus niger*, and *Fusarium solani* [90]. Coating with alginate or tobramycin did not have a significant effect on the bacteriostatic activity of Fe_3_O_4_ NPs against *P. aeruginosa* [84].

Conjugation with polyethylene glycol (PEG) and chitosan allows not only improving the antimicrobial properties of IONPs but also reducing the undesirable adsorption of IONPs on liver macrophages [133,134].

One of the methods for improving the antimicrobial properties is the use of a combination of “green synthesis” and a change in the NP compositions—for example, the addition of gold. The bacteriostatic effect of the mixture *Urtica*/α-Fe_2_O_3_●Ag NPs against *S. aureus*, *Bacillus* sp., *Klebsiella* sp., and *E. coli* was higher compared to *Urtica*/α-Fe_2_O_3_ NPs. An increase in the inhibition zone was proportional to the silver concentration in the composite. Both *Urtica*/α-Fe_2_O_3_●Ag NPs and *Urtica*/α-Fe_2_O_3_ NPs had more pronounced effects on the growth of the Gram-negative strains [36]. Some of the mechanisms of action of Fe_3_O_4_ and Ag NPs are membrane damage, a decrease in the redox potential, and H^+^ fluxes, which lead to the inhibition of the activity of bacterial F_o_/F_1_-ATPase [131].

The combined use of NPs from iron oxides and gold does not reduce the growth of the bacterial biomass of the *E. coli* culture but prevents bacterial cell division [75]; as a consequence, *E. coli* alters their morphology from rods to filaments with a length of several micrometers. The mixture of Fe_3_O_4_ and Au NPs inhibits the growth of the kanamycin-resistant *Escherichia coli* and *Salmonella typhimurium* strains more effectively than Fe_2_O_3_ NPs [75]. 

An approach to an improvement in the antimicrobial properties of IONPs by their conjugation with antibiotics is described by the example of gentamicin [79]. With that, a more pronounced bacteriostatic effect was achieved against Gram-positive *B. subtilis* and *S. aureus* than Gram-negative *E. coli* and *P. areuginosa*. A conjugation with gentamicin reduced the minimum inhibitory concentration against all indicated strains by more than ten times [79]. In several cases, the conjugation of IONPs with antibiotics can give an opposite result. IONPs conjugated with amoxicillin enhanced the growth of *Pseudomonas aeruginosa* and *Staphylococcus aureus* [86]. The presence of organic acids (humic acid) additionally accelerates bacterial growth. In general, it is possible to significantly influence the antimicrobial activity of IONPs by additives, coatings, and conjugates, which, undoubtedly, can be promising in the development of this direction.

### 2.7. Biocompatibility of IONPs

It is shown that IONPs have good biocompatibility and biodegradability. In particular, the intravenous injection of 0.8 mg/kg of γ-Fe_2_O_3_ NPs did not influence the weight gain in rats or cause the activation of apoptosis in HUVEC cells [61]. After intravenous injection, NPs were found in rat lungs, liver, and kidneys but not in the brain or heart. A significant proportion of NPs was eliminated with urine after 72 h [61]. In general, IONPs show an absence or low cytotoxic effects on cell cultures. For example, no adverse effect of IONPs coated with polyethyleneimine, dimercaptosuccinate, or citrate on primary rat cerebellar cortex astrocytes and cultured murine astrocytes was observed [62,135]. IONPs conjugated with PEG-phospholipids (WFION) did not influence the viability of the B16 F10 cell line at doses up to 0.75-mg Fe/mL [136]. Fe_3_O_4_ NPs show a bacteriostatic effect and, at the same time, do not exert a hemolytic action [66,70]. In several cases, IONPs enhance Casp3-dependent apoptosis in HUVEC cells, cause ROS generation, membrane damage, changes in the cytoskeleton, and so on [137]. In general, the cytotoxic properties of IONPs are manifested at much higher concentrations than the antimicrobial properties.

### 2.8. Disadvantages of IONPs

The disadvantages of IONPs include relatively weak antimicrobial action against several strains and insufficient biocompatibility with eukaryotic cells. For example, Fe_2_O_3_ NPs inhibit the growth of *Escherichia coli*, *Staphylococcus aureus*, *Pseudomonas aeruginosa*, and *Bacillus subtilis* less effectively than ZnO and CuO NPs. The inhibitory action of Fe_2_O_3_ NPs on *Escherichia coli* growth was lower than that of ZnO and CuO NPs [138]. This effect may be explained by differences in the antibacterial properties of considered metals. Iron Fe^2+^ is necessary for the proliferation of bacteria [31]. Fe^3+^ inhibited *E. coli* growth in concentrations above 0.25–1 mM, but Fe^3+^ had only a bacteriostatic effect without bactericidal action [139]. Zn^2+^ and Cu^+^ decrease the viability of *Staphylococcus aureus* and *Escherichia coli* in concentrations of 2.41 and 0.46 mM, respectively [140]. Fe NPs exert more pronounced bacteriostatic actions against *Pseudomonas aeruginosa* than Fe_3_O_4_ NPs [84]. Lee et al. [97] did not observe the bactericidal action of Fe_3_O_4_ NPs against *E. coli* contrary to Fe NPs, Ag NPs, or FeSO_4_ NPs. Fe^2+^ from IONPs in the presence of humic acid can enhance the growth of *Pseudomonas aeruginosa* [86]. The bacteriostatic action of Fe_3_O_4_ NPs against various microbial species differs significantly [8]. Fe_3_O_4_ NPs effectively inhibits the growth of *Staphylococcus epidermidis*, *Staphylococcus aureus*, *Bacillus licheniformis*, and *Bacillus subtilis.* The effect is comparable with the action of neomycin. With that, Fe_3_O_4_ NPs are two times less effective at inhibiting the growth of *Bacillus brevis* and *Vibrio cholerae* than neomycin and absolutely do not influence the growth of *Shigella flexneri* and *Pseudomonas aeruginosa.*

Unfortunately, IONPs has not only bacteriostatic and bactericidal activities but toxicity for some eukaryotic cell lines [108]. The main mechanism of IONP toxicity is the production of ROS, which leads to increasing the level of lipid peroxidation, decreasing the antioxidant enzymes, and protein aggregation [141,142,143,144]. IONPs can lead to cell iron overload. Iron overload causes serious deleterious and leads to cell death [142,143]. In addition, a high dose of IONPs increases the lipid metabolism, the breakage of iron homeostasis, and exacerbates the loss of murine liver functions in vivo [145].

The IONP applications in biomedicine are limited due to a lack of control and prediction of the final IONP properties, such as IONP interactions with cells [146]. An important aspect of IONPs in biomedical applications is their surface chemistry [147]. The coating of IONPs by PEG reduces protein adsorption, increases stability to the IONPs, decreases the IONP uptake by culture cells in and by entire organisms in vivo, and increases IONP retention times in the blood flow [148,149,150]. Unfortunately, PEG can be oxidized by host enzymes, which leads to a loss of some PEG-IONP proteins [148]. Proteins are commonly the first biomolecules that IONPs encounter when they interact with biological systems in vitro or in vivo [146]. IONPs may be coated by bovine serum albumin (BSA) or fetal bovine serum [151]. BSA forms a protective layer on the NPs to improve the biocompatibility and transport of the IONPs. BSA-coated IONPs allow to accumulate the drug in the tumor due to an enhanced permeability and retention and to reduce the risk of hypersensitivity reactions [152]. Drugs released from BSA-coated IONPs can be triggered by protease digestion in target tissues, and finally, the unfolding BSA protein on the IONPs can facilitate their clearance by phagocytes after drug delivery [153]. Additionally, BSA coating supports the colloidal stability of the IONPs in cell culture experiments [151]. Multiple specialized characterization methods are widely used to characterize IONP surfaces: TEM, UV-visualization, MD simulation, isothermal titration calorimetry, ζ-potential measuring, etc. [151]. The antibacterial properties of BSA-IONPs remain unclear.

## 3. Conclusions

IONPs have found wide applications in different fields of biomedicine. The antibacterial activities of IONPs are of special interest. However, the situation with the antimicrobial activities of IONPs is ambiguous. On the one hand, the antibacterial activities of IONPs depend, to a significant extent, on the microbial strain, and the inhibitory actions of IONPs are often less pronounced than that of NPs of other metal oxides (CuO or ZnO). On the other hand, IONPs show less-pronounced cytotoxic properties and better biocompatibility in vivo compared to CuO or ZnO NPs. We assume that, in the near future, IONPs will allow achieving a balance between antimicrobial actions and biocompatibility in vivo. In this case, IONPs can be considered potential antimicrobial agents of the new generation. Based on the analyzed data, we believe that the most promising method for increasing the antimicrobial properties of IONPs and improving biocompatibility is “green synthesis” and other variants of the additive or composite generation of nanoparticles.

## Figures and Tables

**Table 1:** List of the microorganisms susceptible to the toxic action of IONPs.

Group of Microorganism	Species/Serotype	Reference
Gram-negative bacteria	*Escherichia coli*	[33,66,67,68,69,70,71,72,73,74,75,76,77,78,79,80]
	*Klebsiella pneumoniae*	[70,72,74,77,80,81,82]
	*Klebsiella* sp.	[36]
	*Proteus mirabilis*	[73]
	*Proteus vulgaris*	[83]
	*Pseudomonas aeruginosa*	[71,79,80,82,84,85,86]
	*Salmonella enterica* serotype *typhimurium*	[76,81,87]
	*Serratia marcescens*	[32,71]
	*Vibrio cholerae*	[8]
	*Xanthomonas* sp.	[83]
Gram-positive bacteria	*Bacillus brevis*	[8]
	*Bacillus cereus*	[87]
	*Bacillus licheniformis*	[8]
	*Bacillus* sp.	[36]
	*Bacillus subtilis*	[8,70,72,73,76,78,79]
	*Corynebacterium* sp.	[75]
	*Enterococcus hirae*	[66]
	*Listeria monocytogenes*	[71,87]
	*Micrococcus luteus*	[67]
	*Staphylococcus aureus*	[8,33,36,67,68,69,70,72,74,76,77,79,80,81,85,86]
	*Staphylococcus epidermidis* *Streptococcus mutans*	[8,88,89]
Fungi	*Aspergillus niger*	[90]
	*Candida albicans*	[87,90]
	*Candida glabrata*	[87]
	*Candida glochares*	[87]
	*Candida saitoana*	[87]
	*Fusarium solani*	[90]

**Table 2:** Parameters of the nanoparticles reported in the literature.

№	Synthesis Method	Composition	Size, nm	Shape	Concentration	Medium, Conditions	Microorganism	Biological Effect	Ref
1	Coprecipitation method	Fe_2_O_3_	25–40	Sph	10–50 µg/mL	NA, 48 h, 37 °C	*E. coli,* *S. aureus,* *S. dysentery*	BS	[33]
2	Chemical precipitation using *Psidium Guajava* leaf extract as a reducing agent followed by heat treatment	Fe_2_O_3_	34	Sph	20–100 µg/mL	MHA, 24 h, 37 °C	*E. coli,* *S. aureus*	BS	[68]
3	Chemical precipitation using *Punica granatum* peel extract as a reducing agent followed by heat treatment	-	-	-	31 µg/mL	MHA, 24 h, 37 °C	*P. aeruginosa*	BS	[70]
4	Wet chemical method	Fe_3_O_4_	33–40	Sph	25–100 µg/mL	NA, 24 h, 37 °C	*E. coli,* *P. vulgaris,* *S. aureus,* *Xanthomonas* sp.	BS	[83]
5	Modified coprecipitation method	Fe_3_O_4_	10.64 ± 4.73	Sph	50–500 µg/mL	NA, 24 h, 3 °C	*E. coli,* *E. hirae*	BS	[66]
6	Coprecipitation	α-Fe_2_O_3_/Co_3_O_4_ composite	25	Rod/ hexag	400–800 µg/mL	MHA, 24 h, 37 °C	*B. subtilis,* *E. coli,* *S. aureus,* *S. typhimurium.*	BC	[76]
7	Chemical precipitation using *Cynometra ramiflora* extract as a reducing agent	Fe_2_O_3_/Fe_3_O_4_	-	Sph	70 µL of IONPs suspension/disk	NA, 24 h, 37 °C	*E.coli,* *S. epidermidis*	BS	[88]
8	Coprecipitation method	α-Fe_2_O_3_, ZnO/α-Fe_2_O_3_	~30	Sph/oval	400–800 µg	MHA, 24 h, 37 °C	*B. subtilis,* *E. coli,* *S. aureus,* *S. typhimurium*	BS	[76]
9	Coprecipitation method	Fe_3_O_4_	6–9	Sph	32–128 μg/mL	LB broth, 37 °C	*E. coli,* *L. monocytogenes,* *P. aeruginosa,* *S. marcescens*	BS	[71]
10	Chemical precipitation using *Sida cordifolia* as a reducing agent and stabilizer	Fe_2_O_3_	16	Sph	50 μg/mL	MHA, 24 h, 37 °C	*B. subtilis,* *E. coli,* *K. pneumoniae,* *S. aureus*	BS	[119]
11	Coprecipitation method	IONPs with amoxicillin	-	-	0.05–10 mM	TSB, 24 h, 37 °C	*P. aeruginosa,* *S. aureus*	Stimulation of bacterial growth in the presence of humic acid	[86]
12	Ready commercial product (Sigma-Aldrich)	Fe_2_O_3_	<5	-	0.05–10 mM	LB, 37 °C	*E. coli*	BC	[99]
13	Coprecipitation using the aqueous extract of corn (*Zea mays* L.) ear leaves	Fe_3_O_4_	37.86	Sph	25–50 μg/disc	NB, 37 °C at 24 h, for bacteria, PDA, 28 °C at 48 h for fungi	*B. cereus,* *C. albicans,* *C. glabrata,* *C. geochares,* *C. saitoana,* *E. coli,* *L. monocytogenes,* *S. aureus,* *S. typhimurium,*	BS	[87]
14	Coprecipitation method in alkaline media with leaf extract of *A. mexicana*	Fe_3_O_4_	10–30	Sph	12.5–50 mg/disc	MHB, 24 h, 37 °C	*B. subtilis,* *E. coli,* *P. mirabilis,*	BS	[73]
15	Laser ablation in dimethylformamide (DMF) and sodium dodecyl sulfate (SDS) solutions	α-Fe_2_O_3_	50–110	Sph	4.25 mg/mL	NA, 24 h, 37 °C	*E. coli,* *P. aeruginosa,* *S. aureus,* *S. marcescens*	BS	[91]
16	Coprecipitation using *Couroupita guianensis* aqueous fruit extract	Fe_3_O_4_	~17	Sph	25–75 μg/mL	NA, 24 h, 37 °C	*E. coli,* *K. pneumoniae,* *S. typhimurium*	BS	[81]
17	Coprecipitation	Fe_3_O_4_ coated by SiO_2_	~20	Sph	-	NA, 24 h, 37 °C	*E. coli,* *S. aureus,*	BS	[130]
18	Chemical precipitation using *Tridax procumbens* leaf extract as a reducing agent	Fe_3_O_4_	-	Sph	10–40 μL	PDA	*P. aeruginosa*	BS	[120]
19	Coprecipitation	Fe_3_O_4_	8	Sph	50–200 μg/mL	LB, 37 °C, 14 h	*E. coli*	BS	[75]
20	Ultra-large-scale synthesis	Fe_3_O_4_ or Fe_3_O_4_ coated by alginate	~16, for coated with alginate ~230	Sph	2.5–10 μg	LB, 37 °C, 16–18 h	*P. aeruginosa*	BS	[95]
21	Chemical precipitation using *Ruellia tuberosa* leaf aqueous extract as a reducing agent	FeO	52.78	Rod	25–75 μg/mL	MHA, 24 h, 37 °C,	*E. coli,* *K. pneumoniae,* *S. aureus*	BS	[74]
22	Coprecipitation	PEG-Fe_3_O_4_	26 ± 1.26	Sph	0.1–100 μg/mL	-	*E. coli,* *M. luteus,* *S. aureus,*	BS	[67]
23	Coprecipitation using *Malva sylvestris* as a reducing agent	Fe_3_O_4_	30–50	Sph	62.5 mg/mL	BHI, 24 h, 37 °C,	*Corynebacterium* sp., *K. pneumonia,* *P. aeruginosa,* *S. aureus,*	BS, BC	[82]
24	One-pot hydrothermal method	Fe_3_O_4_	~160	Sph	300–1000 μg/mL	LB, 37 °C, 14 h	*E. coli,* *S. aureus*	BS	[69]
25	Chemical precipitation using orange peel extract as a reducing and stabilizing agent	Fe_2_O_3_	~50	-	0.5 mg/mL	NA, 36 °C, 24 h	*B. subtilis,* *E. coli,* *P. aeruginosa,* *S. aureus*	BS	[121]
26	Chemical precipitation using *Urtica* leaf extract as a reducing agent	α-Fe_2_O_3_, α-Fe_2_O_3_-Ag	100–200	Different	35 µg/mL 5–35 μg/disc	MHA, 24 h, 37 °C,	*Bacillus sp.,* *E. coli,* *K. pneumoniae,* *S. aureus*	BS	[36]
27	Coprecipitation	Fe_3_O_4_	10.64 ± 4.73	Sph	50–250 μg/mL	Peptone medium, 24 h, 37 °C,	*E. coli DH5α*-pUC18 ampicillin-resistant; *E. coli* pARG-25 kanamycin-resistant	BS	[66]
28	Coprecipitation	Fe_3_O_4_	10–120	Sph	50 mg/mL	NA, 24 h, 37 °C,	*B. brevis,* *B. licheniformis,* *B. subtilis,* *E. coli,* *P. aeruginosa,* *S. aureus,* *S. epidermidis,* *S. flexneri,* *V. cholera*	BS	[9]
29	Coprecipitation	Fe_3_O_4_, Co/Fe_2_O_4_, Mn/Fe_2_O_4_	14–68	Cubic spinel	25–2000 μg/mL	NB, NA, 24 h, 37 °C,	*B. subtilis,* *E. coli*	BS	[102]
30	Solvothermal method	IONPs modified with oleic acid	75–1110	Sph	25–125 μg/mL	LB broth, 48 h, 37 °C,	*P. aeruginosa,* *S. aureus*	BS	[85]
31	Laser ablation in dimethylformamide (DMF) and sodium dodecyl sulfate (SDS) solutions	α-Fe_2_O_3_	50–110	Sph	-	NA, 24 h, 37 °C,	*E. coli,* *P. aeruginosa,* *S. aureus,* *S. marcescens*	BS	[91]
32	Sol–gel combustion	Fe_2_O_3_	35.16 ± 1.47	Sph	65 ± 1.5 μg/mL	MHB, 24 h, 35 ± 2 °C,	*B. subtilis,* *E. coli,* *P. aeruginosa,* *S. aureus*	Low BC	[13]
33	Matrix-mediated method using PVA (polyvinyl acetate)	Fe_3_O_4_/Fe_2_O_3_	9 ± 4	Sph	30–3000 μg/mL,	TSB, 24 h, 37 °C,	*S. aureus*	BS, BC	[32]
34	Laser ablation in the water	IONPs/carbon nanotubes	6–7	Sph IO on the carbon nanotubes	400–800 μg/mL	NB, 24 h, 37 °C,	*E. coli,* *K. pneumoniae,* *S. aureus*	BS	[77]
35	Coprecipitation	Fe_3_O_4_ conjugated with TEPSA or TPED	14.6 ± 1.4, 20.4 ± 1.3 or 21.2 ± 1.6	Sph	1–3 μg/mL	TYE, 24 h, 37 °C, in the dark	*Streptococcus mutans*	BC	[89]
36	Coprecipitation	Fe_3_O_4_ coated by citric acid	~30	Sph	100 μg/mL	NA, 24 h, 37 °C,	*E. coli,* *S. typhimurium*	BS	[131]
37	Coprecipitation method	Fe_3_O_4_, Fe_2_O_3_ coated by chitosan	10–20	Sph	2.5–50 μM	NB, 37 °C	*B. subtilis,* *E. coli*	BC	[78]
38	Coprecipitation	Fe_3_O_4_ coated by chitosan	~11	Sph	30–40 μg/mL	TSA for bacteria, YEPD for *C. albicans*, CYA for *A. niger*, Potato sucrose agar for *F. solani.* 48 h at 30 °C	*A. niger,* *B. subtilis,* *C. albicans,* *E. coli,* *F. solani*	BS	[90]
39	Coprecipitation method	Fe_2_O_3_, FeO, coated by gentamicin	10–15	Sph	200 µg/mL	LB broth, 24 h, 37 °C	*B. subtilis,* *E. coli,* *P. aeruginosa,* *S. aureus*	BC	[79]
40	Coprecipitation	Fe_3_O_4_	20–25	-	5–80 μg/mL	NB, 24 h, 37 °C	*B. cereus,* *K. pneumoniae,*	BS, BC	[132]
41	Coprecipitation using *Glycosmis* *mauritiana* water extract as a reducing agent	Fe_3_O_4_	<100	Sph	10–30 µg/µL	MHA, 24 h, 37 °C,	*E. coli,* *K. pneumoniae,* *P. aeruginosa,* *S. aureus*	BS	[80]

BHI—Brain heart infusion, BS—bacteriostatic effect, BC—bactericidal effect, Hexag—hexagonal, IONPs—iron oxide nanoparticles, LB—lysogeny broth, MHA—Mueller–Hinton Agar, NA—Nutrient Agar, NB—Nutrient broth, PDA—Potato dextrose agar, Rod—rod-shaped, Sph—spherical, TEPSA—3-(triethoxysilyl) propylsuccinic anhydride, TPED—N-[3-(trimethoxysilyl)propyl] ethylenediamine, TSB—Tryptic soy broth, YEA—Czapek yeast extract agar, and YEPD—yeast extract peptone dextrose.

## Data Availability

The raw data supporting the conclusions of this article will be made available by the authors without undue reservation.
